# Patient Shielding in Computed Tomography: Analysis of Influencing Factors and a Conceptual Framework for Decision-Making

**DOI:** 10.3390/healthcare14172818

**Published:** 2026-09-02

**Authors:** Yating Qiao, Hui Liu, Qiang Zhou, Fei Tuo

**Affiliations:** China CDC Key Laboratory of Radiological Protection, National Institute for Radiological Protection, Chinese Center for Disease Control and Prevention & Chinese Academy of Preventive Medicine, Beijing 100088, China

**Keywords:** computed tomography (CT) scan, patient shielding, conceptual framework, radiation protection

## Abstract

**Background:** Although lead shielding during computed tomography (CT) examinations has served as routine clinical practice, its actual efficacy and execution remain controversial, showing noticeable divergences across national policies and international clinical guidelines. This discrepancy highlights a disconnect between clinical habits and evolving technical evidence. **Methods:** This narrative review examines the operational, institutional, legal, and cognitive constraints sustaining these divergent practices. **Results:** Rather than offering empirical data, this work synthesizes existing literature to propose a preliminary conceptual framework integrating these multi-dimensional factors. **Conclusions:** Unlike prior studies focusing narrowly on dosimetric outcomes, this framework incorporates transitional stages in CT technology and institutional capacities, offering a scalable, context-specific perspective to inform professional discussions and support localized evaluations regarding radiation protection.

## 1. Introduction

Radiation safety has become increasingly important as the use of computed tomography (CT) has risen dramatically worldwide, accounting for over 60% of medical radiation exposure [1,2,3,4]. In this context, the Linear No-Threshold (LNT) model and the As Low As Reasonably Achievable (ALARA) principle have prompted a re-evaluation of the scientific rationale for routinely shielding non-scanned body parts, a practice inherited from early radiography that persists in clinical routine [5,6].

The LNT model, recommended by the International Commission on Radiological Protection (ICRP) as a prudent foundation for regulatory control, assumes a proportional relationship between radiation dose and cancer risk without a threshold [6]. In a review of relevant epidemiological studies, NCRP (2018) concluded that current epidemiological data support the continued use of the LNT dose–response relationship for radiological protection purposes, with no other model representing a more pragmatic or prudent interpretation [7]. However, its application to low-dose diagnostic CT remains debated. A primary limitation is the reliance on risk extrapolation from high-dose data, such as atomic bomb survivor cohorts, which introduces uncertainty into low-dose risk estimation [8,9,10]. Nevertheless, it should be recognized that while low doses may be measured or estimated with reasonable reliability, the associated cancer risk is uncertain, and becomes increasingly uncertain as dose decreases. Radiobiological studies indicate that doses below 100 mGy show no direct, definitive harmful effects and may trigger adaptive responses such as enhanced DNA repair [11,12,13,14]. Conversely, epidemiological investigations report associations between low-dose exposure and elevated cancer risks [15,16,17,18,19,20,21], though these observational findings are subject to confounding and reverse causality. Consequently, while the true biological risks of low-dose radiation remain a subject of active debate [22,23,24], the LNT-based approach continues to serve as a pragmatic and ethically defensible framework under prevailing uncertainty.

The ALARA principle is central to patient radiation safety, and modern CT technology has made accurate dose management possible through automated exposure control, iterative reconstruction, and targeted scan localization. Studies have demonstrated that low-dose CT scanning reduces radiation exposure by 66.1% among pediatric patients with lymphoma while preserving full diagnostic accuracy [25]. Conventional lead shielding typically offers negligible clinical protection and may inadvertently compromise image quality or increase patient dose for two main reasons: (1) radiation exposure outside the imaging field is primarily caused by internal scatter within the patient’s body, making the external shielding effect clinically insignificant; and (2) anatomic differences among patients make it challenging for operators to precisely position shielding devices without interfering with sensitive organs, such as the thyroid and gonads, or affecting scan parameters. This method commonly disrupts automatic exposure control systems, resulting in unexpected increases in scan dose, artifacts obscuring essential anatomical structures, or even repeat scans [26,27,28,29]. For example, adding a lead apron outside the imaging field provides no meaningful dose decrease during juvenile chest CT [30].

Global patient radiation protection procedures have undergone a significant paradigm shift driven by advances in scientific knowledge and the accumulation of clinical data [31]. In China, as in several other countries, routine patient shielding remains standard practice in diagnostic radiology, guided by domestic regulations such as the Requirements for Radiological Protection in Diagnostic Radiology (GBZ 130 2020) [32] and the Radiological Health Protection Standards for Examinees Undergoing Medical X-ray Diagnosis (GB 16348 2010) [33]. However, with the improved dose-control capabilities of modern imaging technology and thorough evaluations of the actual protective value of shielding, European and American nations have steadily moved toward de-shielding [5,31]. The American Association of Physicists in Medicine (AAPM, 2019) [34], UK Health Security Agency (UKHSA, 2020) [35], American Dental Association (ADA, 2024) [36], and the Gonad and Patient Shielding (GAPS) Group [37] have released pertinent position papers in recent years. According to these documents, the routine use of shielding, such as lead aprons, during most traditional radiography is no longer advised because it has no therapeutic value and may interfere with automatic exposure management and increase the risk of repeat exposure. This divergence may reflect varying rates of adopting scientific evidence, as well as institutional, traditional, and policy factors, etc.

To explore this issue, we conducted a narrative review of PubMed through 5 September 2025, using the search string ((“patient shielding” OR “patient radiation shielding” OR “radiation protection shielding” OR “contact shielding” OR “protective apron” OR “lead shielding”) AND (CT OR “computed tomography”)), limited to English-language original articles and reviews in clinical medicine and radiation protection. All retrieved records were imported into EndNote for deduplication. Titles and abstracts were first screened by the primary author to exclude irrelevant studies, followed by a full-text review conducted jointly with the co-advisors to determine final inclusion based on relevance to CT shielding. Through qualitative synthesis of the selected literature, recurring themes were iteratively grouped to derive the four categories of influencing factors analyzed in this study. This study has two primary objectives: (1) to examine the cognitive, institutional, and operational drivers of the conflict over routine CT patient shielding, and (2) to propose a preliminary, hypothetical conceptual framework for decision-making considerations in shielding practices. Intended solely as an exploratory theoretical reference rather than a clinical protocol, this framework provides a preliminary basis for radiation safety discussions, subject to future practical evaluation and optimization.

## 2. Influencing Factors of Patient Shielding

### 2.1. Cognitive and Psychological Factors

Cognitive patterns and psychological factors play a notable role in shaping the adoption or discontinuation of patient shielding, operating across three primary dimensions:

First, variations in the dissemination of professional knowledge influence clinical practice. Continuing education programs do not always reflect the latest updates in CT dosimetry and risk communication at uniform rates, contributing to differing interpretations of modern safety guidelines among practitioners [38,39].

Second, perceptions of radiation risk diverge among the public and clinicians. While awareness of radiation safety has generally increased, translating complex and sometimes conflicting low-dose risk data into intuitive clinical understanding remains challenging [40,41,42,43]. Concerns about potential risks—influenced by varying interpretations of biological effects—can lead both patients and providers to weigh the psychological comfort of visible protective measures against quantitative estimates.

Third, behavioral habits and established workflows naturally shape clinical routines. Even when new evidence is acknowledged, modifying long-standing practices requires operational adjustments. Clinicians frequently balance traditional safety protocols with evolving standards, resulting in diverse implementation paces across institutions.

### 2.2. Systemic and Institutional Barriers

Variations in patient shielding practices across different healthcare systems are also shaped by broader systemic and institutional frameworks, rather than individual choice alone. These include regulatory standards, operational structures, and resource allocation mechanisms that vary globally.

Regional inequalities in resource allocation and equipment infrastructure influence local implementation. According to data from the European Association of Imaging Equipment Manufacturers [44], there is a significant global disparity in CT unit density and modernization: the EU 27 (28.2 units per million people) is much higher than Russia (19.5), Greater China (14.7), Brazil (12.1), and India (3.9). Additionally, over 21% of CT scanners in Europe have been in use for more than 10 years. These global disparities highlight diverse technological environments across healthcare systems, which naturally shape how local institutions approach equipment capabilities and technical protocols.

Second, clinical efficiency and utilization pressures influence institutional priorities. Studies indicate that optimizing imaging utilization remains a widespread global challenge, with instances of unindicated or repeated CT scans reported across various settings [45,46,47,48,49]. While substantial economic impacts are associated with unnecessary imaging globally—such as estimated annual costs in the United States, where CT contributes significantly to the population radiation burden [50,51]—these broader efficiency and workload pressures mean that healthcare systems must balance fundamental operational demands alongside the implementation of specialized radiation safety policies, contributing to varied institutional pacing in clinical practice adjustments.

Finally, institutional priorities and workflow demand shape the pace of clinical adjustments. Healthcare organizations navigate multiple operational pressures, including administrative workflows, resource distribution, and patient throughput. These systemic considerations guide how institutions balance traditional safety measures with emerging recommendations in their daily practice.

### 2.3. Legal and Ethical Barriers

Clinical decision-making regarding patient shielding is further shaped by the complex interplay between evolving scientific evidence and established legal frameworks.

A central legal challenge stems from the temporal gap between emerging technical guidelines and the legal standard of care. In medical litigation, standards are typically anchored in statutory regulations, authoritative textbooks, and prevailing clinical traditions rather than the most recent academic findings [52]. Consequently, clinicians often must balance scientific modifications against potential liability concerns, occasionally fostering a cautious or defensive medical approach during periods of practice transition [53,54,55,56,57,58]. In the absence of explicit regulatory alignment, practitioners may choose to maintain conventional shielding to mitigate legal exposure, regardless of updated technical recommendations.

Ethically, this environment complicates patient communication and shared decision-making. Discussing the modification or omission of traditional protective measures requires conveying complex low-dose risk data while managing patient expectations. When legal benchmarks lag behind scientific updates, practitioners face a dual challenge: upholding the ethical principle of transparent disclosure while navigating the legal vulnerabilities associated with deviating from established practice.

### 2.4. Operational and Alternative Solutions Barriers

Finally, the transition of patient shielding practices encounters practical hurdles related to operational complexity and the availability of alternative communication tools. Modifying long-standing protective protocols involves more than a technical directive; it requires a systematic restructuring of clinical workflows and patient communication strategies.

From an operational perspective, redesigning clinical processes entails implementation costs and logistical hurdles. Radiology departments operating under heavy workloads frequently manage diverse equipment models and high patient volumes. In such environments, altering established routines requires comprehensive staff training, workflow adjustments, and resource support. Without explicit institutional frameworks or efficiency support mechanisms, any procedural change that temporarily affects throughput can face operational hesitation at the departmental level.

Furthermore, practical challenges arise from the need for effective alternatives to physical shielding. Historically, lead aprons and similar devices have served not only physical functions but also psychological roles as visible symbols of safety for patients [59]. While modern CT systems deliver significantly reduced radiation doses, public concerns regarding radiation safety persist [43,60]. Consequently, removing physical shielding without standardized alternative communication tools can increase patient anxiety and place substantial demands on clinicians’ time. Developing reliable, non-shielding communication and education paradigms thus remains a practical consideration for clinical implementation.

## 3. A Proposed Conceptual Framework for Optimizing Patient Shielding Practices

To explore the transition toward evidence-based radiation protection, we propose a preliminary three-step conceptual framework designed to bridge clinical decision-making, operational execution, and ongoing evaluation (Table 1). The validity and practical utility of this model remain to be tested through future empirical implementation.

**Table 2:** CT Equipment Classification and Balanced Considerations.

Equipment Classification	Technical Features	Balanced Considerations
Class A	Contemporary systems equipped with 3D real-time automatic exposure control (AEC), tube current modulation (TCM), and advanced iterative reconstruction algorithms.	While modern system-level dose optimization (TCM and algorithms) largely governs overall patient exposure, the potential interaction between external contact shields and real-time AEC algorithms remains a primary technical consideration. Decision-making in this tier typically involves weighing the limited dosimetric benefit against algorithmic behavior (such as potential over-compensation) and workflow realities.
Class B	Systems featuring conventional 1D/2D AEC and basic or hybrid iterative reconstruction (IR) technologies.	In this transitional tier, traditional AEC response to superficial attenuators and the dominance of internal patient-scattered radiation present ongoing technical challenges. Clinical practices often balance the desire for organ-specific adjunct protection against the physical limitations of surface shielding within conventional protocols.
Class C	Legacy or basic systems lacking advanced AEC, typically operating with fixed tube current parameters and conventional filtered back projection (FBP).	Operating without dynamic tube current modulation, legacy platforms present a scenario where surface shields may theoretically attenuate direct primary radiation for superficial organs. However, practitioners must carefully weigh this theoretical surface interception against unmitigated internal scatter and the priority of overarching technical parameter optimization.

Step 1: Enhancing Clinical Decision-Making

This tier prioritizes foundational appropriateness over routine habits. By integrating structured risk-benefit assessments and review mechanisms, the framework aims to assist clinicians in verifying diagnostic indications, minimizing redundant imaging, and balancing medical objectives with patient-centered considerations prior to scanning.

Step 2: Optimizing the Scanning Process via Technical Stratification

This tier translates decisions into practice by addressing equipment capabilities and workflow execution. As detailed in the three-tier classification of Table 2, the framework categorizes equipment based on two primary technical determinants: the sophistication of automatic exposure control (AEC) and tube current modulation (TCM), and the generation of reconstruction algorithms [61,62].

The underlying rationale for this classification rests on dosimetric interactions: while legacy systems without advanced AEC generally experience minimal tube output alteration from external attenuators, contemporary systems with 3D real-time AEC may interpret superficial shields as part of the patient, potentially increasing tube current and offsetting algorithmic dose reductions. Crucially, this three-tier schema is proposed herein strictly as an exploratory conceptual framework—rather than a validated clinical standard—intended to provide a qualitative structural basis for local safety discussions rather than prescriptive operational rules.

Step 3: Implementing Evaluation and Continuous Improvement

This tier outlines a preliminary mechanism for quality monitoring and protocol refinement. Through phased pilot implementation and a closed-loop evaluation system tracking compliance, image quality, and feedback, institutions can iteratively assess local protocols to explore sustainable practice adaptation.

## 4. Conclusions and Perspectives

In summary, this review outlines several factors that may influence clinical decisions regarding patient shielding in CT imaging. Building upon these considerations, we outline a preliminary, three-step conceptual framework intended to serve as a reference point for departmental discussions. This proposal is strictly exploratory and aims to provide a descriptive reference rather than a definitive guideline.

Despite ongoing discussions regarding a paradigm shift in radiological patient shielding, clinical implementation remains challenging. A multi-center survey across 225 centers in 35 countries indicated that contact shielding is still widely utilized, predominantly in conventional radiography where the gonads are the primary shielded organs, followed by the thyroid, female breasts, and eye. While 83.6% of respondents reported they would follow guidance if issued by major European bodies, communicating and implementing these policies remains challenging [63]. An evolving development in this debate is the gradual shift toward unshielded protocols [5]. A systematic literature review evaluating the benefits and drawbacks of patient shielding found that dose savings were rather minimal [64]. For instance, following the March 2020 recommendations by the British Institute of Radiology (BIR) and five other organizations, the American Academy of Oral and Maxillofacial Radiology (AAOMR) and the American Dental Association (ADA) issued updated guidelines in September 2023 and April 2024, respectively, outlining explicit positions on shielding [65]. Central to this ongoing debate is the persistent lack of robust empirical data establishing safe radiation dose thresholds. An investigative study quantifying scatter radiation during CT scans has shown that contact shielding can reduce scatter to out-of-field organs—lowering doses by 74–75% for the eyes, 47–50% for the thyroid, and 56% for the breasts relative to unshielded conditions [66]. Additionally, whether the increasing utilization of CT imaging leads to long-term dose accumulation—particularly exposure to radiosensitive organs—and potentially induces stochastic effects such as radiation-induced malignancies remains to be studied. Consequently, deciding whether to utilize patient shielding remains a complex and cautious matter, highlighting the need for ongoing research and optimized protocols to support this clinical process.

While this exploratory model offers a structured frame of reference for ongoing debates, several notable limitations of this work must be acknowledged. First, the framework remains purely conceptual and lacks real-world clinical validation. It is intentionally broad and omits granular operational details, specific technical parameters, and tailored recommendations for diverse anatomical regions or patient populations. Furthermore, the model does not account for local baseline variations, such as the actual regional distribution, proportion, and performance profiles of legacy versus contemporary CT equipment. Therefore, the practical utility, safety, and broad applicability of this framework remain unproven.

To address these shortcomings, future work must move beyond high-level conceptualization. Subsequent research should prioritize empirical data collection—such as local equipment surveys and multi-center evaluations—to better understand baseline operational realities. Prospective, site-specific studies will be essential to test, refine, and determine whether such structured approaches can be practically and safely integrated into routine clinical workflows.

## Figures and Tables

**Table 1:** An Integration Framework for Changes in CT Radiation Protection Practices.

Implementation Steps	Key Implementation Points	Intended Objectives
Step 1: Enhancing Clinical Decision-Making	Indication Review: Explore integrating electronic health record systems to assist in reviewing examination indications and identifying potential duplicate scans. Risk-Benefit Assessment: Develop structured guidance to assist clinicians in evaluating the clinical necessity of radiological procedures. Contextual Decision Support: Align device technical capabilities (Classes A, B, and C, Table 2) with patient clinical characteristics to inform personalized protective considerations.	Aims to assist in minimizing redundant imaging at the initial stage and establishing a structured basis for personalized examination planning.
Step 2: Improvements to the scanning process	Protocol Standardization: Outline technical parameters, patient positioning, and shielding considerations tailored to different equipment and patient cohorts, supported by staff training. Patient Communication: Utilize structured communication materials to discuss examination necessity, potential radiation risks, and protective measures, aiming to improve patient understanding. Technical Optimization: Monitor system-level features (e.g., automatic exposure control) and track image quality metrics to balance radiation safety with diagnostic efficacy.	Explores pathways to standardize operational execution and balance radiation dose, image quality, and patient preferences.
Step 3: Implement evaluation and improvements	Phased Pilot Exploration: Initiate gradual, flexible pilot implementations under localized protocols, allowing for contextual adjustments based on clinical feedback. Closed-Loop Monitoring: Establish iterative assessment mechanisms tracking operational metrics—including procedural compliance, image quality, radiation dose, and patient experience.	Aims to provide a cyclical framework, though the long-term feasibility and adaptability require future empirical validation.

## Data Availability

No new data were created or analyzed in this study. Data sharing is not applicable to this article.
